# Non-Destructive Spectroscopic Techniques and Multivariate Analysis for Assessment of Fat Quality in Pork and Pork Products: A Review

**DOI:** 10.3390/s18020377

**Published:** 2018-01-28

**Authors:** Christopher T. Kucha, Li Liu, Michael O. Ngadi

**Affiliations:** Department of Bioresource Engineering, McGill University, Macdonald Campus 21,111 Lakeshore Road, Ste-Anne-de-Bellevue, Quebec, QC H9X 3V9, Canada; christopher.kucha@mail.mcgill.ca (C.T.K.); li.liu5@mcgill.ca (L.L.)

**Keywords:** hyperspectral imaging, spectroscopy, multivariate analysis, pork, fat quality, fatty acid, solid fat content, iodine value, oxidative stability, fat colour

## Abstract

Fat is one of the most important traits determining the quality of pork. The composition of the fat greatly influences the quality of pork and its processed products, and contribute to defining the overall carcass value. However, establishing an efficient method for assessing fat quality parameters such as fatty acid composition, solid fat content, oxidative stability, iodine value, and fat color, remains a challenge that must be addressed. Conventional methods such as visual inspection, mechanical methods, and chemical methods are used off the production line, which often results in an inaccurate representation of the process because the dynamics are lost due to the time required to perform the analysis. Consequently, rapid, and non-destructive alternative methods are needed. In this paper, the traditional fat quality assessment techniques are discussed with emphasis on spectroscopic techniques as an alternative. Potential spectroscopic techniques include infrared spectroscopy, nuclear magnetic resonance and Raman spectroscopy. Hyperspectral imaging as an emerging advanced spectroscopy-based technology is introduced and discussed for the recent development of assessment for fat quality attributes. All techniques are described in terms of their operating principles and the research advances involving their application for pork fat quality parameters. Future trends for the non-destructive spectroscopic techniques are also discussed.

## 1. Introduction

Fat quality is a significant factor in pork and pork products quality, having critical correlations to nutrition, sensory characteristics, and shelf-life and safety. For instance, the most notable deleterious impacts of fat quality are the development of rancidity and off-flavors which affect the eating experience, and the high relative levels of oxidative degradation products, which have implications on human health [1]. Despite its impact on pork quality, fat quality has received less attention in the last decades compared to other meat quality attributes such as pH, drip loss, moisture content, tenderness, and color [2]. However, more recently, the pork industry has put more emphasis on fat quality as a measure of pork quality because of the global market competition that requires utilization of specific fat quality parameters. For instance, to produce bacon and sausage, firm fat is the ideal quality parameter required [3]. Besides, in the past, nutritionists have recommended a reduction in the total fat intake to curb the potentially adverse effect of fat on diseases such as obesity and coronary heart diseases arising from the consumption of excess fat, but the recommendations are shifting towards fat quality rather than quantity [4]. Therefore, for the meat industry to provide consumers with high-quality meat and its diverse products, it has the onerous task to determine the quality of the fat in the meat accurately. Thus, there is a heightened interest in fat quality measurement techniques to meet consumer health demands and the technological requirements of the processor.

Traditionally, fat quality evaluation methods such as gas chromatography, iodometric titration, and spectrophotometric methods are widely popular and are mostly applied at the laboratory level for evaluation of fatty acids (FA), iodine value (IV), and oxidative stability of fat, respectively. Apparently, these analytical techniques, though sensitive and straightforward, are too tedious, and time-consuming to stand the demands of the modern meat processing industry. Furthermore, they do not only require expensive chemical solvents but are also destructive hence not suitable for on-line and real-time measurement of fat quality. To address these issues, the pork industry seeks suitable alternative techniques. Thus, meat scientists and food process engineers are posed with the challenge to develop newer alternative techniques which are not only rapid but non-destructive to estimate fat quality quantitatively. Therefore, several non-destructive spectroscopic techniques such as infrared spectroscopy (IR), nuclear magnetic resonance spectroscopy (NMRS), Raman spectroscopy (RS), and hyperspectral imaging (HSI) are being developed for evaluation of fat quality for rapid and online applications.

Non-destructive spectroscopic methods are widely recognized as valuable tools in meat analysis due to the several advantages they offer over the conventional methods such as repeatability, capability for bulk measurements, and their ability to provide multi-constituent analysis of virtually any matrix from a single spectrum. By these advantages, they have gained acceptance within the food industry for raw material testing, product quality control, and process monitoring [5,6,7,8]. Multivariate analysis is instrumental in analyzing NIR spectral data. The significant benefit of multivariate analysis is its ability to reduce the amount of the data sets, to build classification and prediction models, and to enhance the accuracy and robustness of models based on spectral data analysis. Spectral pre-treatment and feature extraction are important in the building robust models. The use of one or both can have a positive influence on the methods’ performance and can lead to potentially simpler solutions if the most informative wavelengths are identified. Pre-treatment techniques mainly including multiplicative scatter correction (MSC), standard normal variate (SNV), smoothing, baseline removal, and first and second derivatives, are used to reduce and correct possible interferences related to scattering, baseline shift, path-length variation, and overlapping bands. Variable selection techniques, such as principal component analysis (PCA), regression coefficient analysis, successive projections algorithm (SPA), uninformative variable elimination (UVE), and genetic algorithms (GA), are commonly used to select the most informative spectral regions or the optimal wavelengths for simplifying the modeling purposes and developing on-line NIR-based multispectral spectrometer detection systems [9]. Commonly used modeling methods for quantitative analysis include multiple linear regression (MLR), partial least squares regression (PLSR), least square support vector machines (LS-SVM), and neural networks (NN). A series of statistical parameters generally evaluates the obtained model such as the determination coefficients of calibration (R^2^_C_), cross-validation (R^2^_CV_) and prediction (R^2^_P_); the corresponding root mean square error estimated by calibration (RMSEC), cross-validation (RMSECV) and prediction (RMSEP); as well as the overall indicator of residual predictive deviation (RPD). A good model should have higher values of R^2^_C_, R^2^_CV_, R^2^_P_ and RPD, and lower values of RMSEC, RMSECV, and RMSEP as well as a small difference between them.

In recent years, several reviews dealing with non-destructive spectroscopic techniques for fat quality analysis in meat have been published. For instance, [10] focused on lipid components and measurement of oxidative deterioration in fish and fish product. Another study was devoted to the applications to some muscle foods [11]. However, even though non-destructive spectroscopic techniques have been widely used for fat quality measurements in pork, no review has been published to address these applications specifically despite the diverse applications of pork fat and its influence on pork quality, and processed products. This paper provides an overview of the traditional fat quality assessment methods with emphasis on IR, NMRS, RS, and HSI to substitute these methods. Also, the review highlights the fundamentals of these techniques and the most recent applications of the spectroscopic methods in tandem with chemometric modeling approaches for different fat quality parameters including FA composition, solid fat content (SFC), IV, color, and oxidative stability.

## 2. Fat Quality Attributes

**Figure 1:**
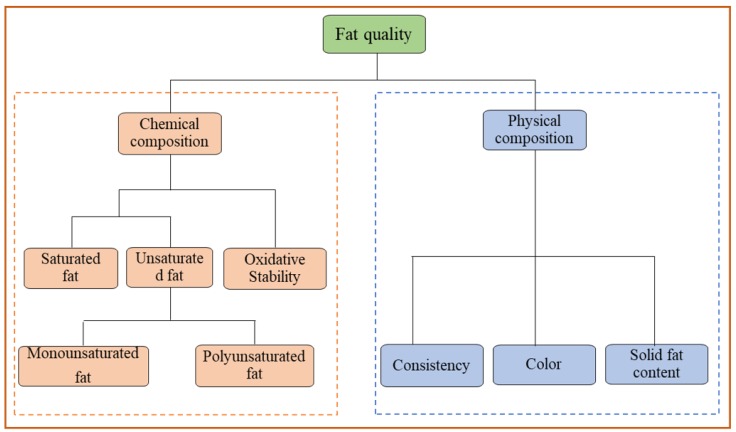
Chemical and physical parameters used to desceibe the quality of fat.

There are various traditional techniques used in the meat processing industry for quality monitoring of pork and pork products. Most of these methods are unsuitable for use in the present processing systems due to the level of their inaccuracies. For instance, in traditional techniques, a limited portion of the muscle is obtained for assessment of an analyte which may not be entirely true representative of that analyte in the muscle, which, therefore, leads to erroneous results. Furthermore, traditional methods are not economical primarily due to the expensive chemical reagents that are employed, the labor that requires highly trained personnel, and the dynamics of the process are often missed because of the time needed to perform the analysis. In addition, fat quality analysis requires instrumentation that is robust and durable enough to stand the harmed environmental humidity commonly encountered in the food processing plants. By that, the meat processing industry needs newer technologies which are cost effective. In fat quality analysis, it is important to assess the physical and chemical quality attributes such as the FA composition, SFC, color, oxidative stability and IV (Figure 1).

### 2.1. Fatty Acids

Fat quality is chemically defined regarding the FA composition, which is commonly expressed as a set of percentages corresponding to the relative content of each FA in the total FAs that have been determined [12]. The FAs are classified per their chemical structure into saturated (SFA) and unsaturated (UFA). UFA could further be monounsaturated (MUFA) or polyunsaturated (PUFA). The proportions of the FAs define the physical properties and differentiate one fat from the others [13]. The SFA are solid at room temperature and have a greater melting point than unsaturated FA (MUFA, PUFA), which contains at least a double bond in their structures. Moreover, as the number of double bonds increases, fat becomes more unsaturated with a decreased melting point and softer consistency at room temperature. The total amount of SFA, MUFA, PUFA and the ratios between FAs, especially SFA:PUFA, are often used to determine technological fat quality with higher levels of saturation indicating more desirable quality and increased unsaturation indicating undesirable fat quality. The characteristics exhibited by fat tissues are as a result of various factors such as feed, sex, breed, and age. These factors are beyond the scope of the present review, but they have been considered by [14,15].

The most common method for determination of FAs is by the gas chromatography (GC) where they are converted into methyl esters to the simplest convenient derivatives [16]. Although GC analysis is time-consuming, regarding the accuracy, reproducibility, and repeatability, it is a remarkable and satisfactory method [17]. With the growing awareness of the importance of a healthy and balanced diet, consumers and human nutritionist need accurate nutritional information on the FA composition of meat [18]. This information is not readily available on the individual cuts in the pork slaughterhouse mainly due to the lack of suitable measurement tools, which lead to highly variable products being marketed without the controlled level of the fat quality [19]. There is, therefore, the need for objective means of measuring FAs in pork considering the high-throughput and productivity in the industry and the unsuitability for routine quality control of the conventional methods, which are evident by high labor cost and hazardous waste generation.

### 2.2. Iodine Value

IV is a measurement to determine the degree of unsaturation present in meat fat by the addition of iodine. Since unsaturated FAs yield fat that is soft, IV can be used as an indicator of fat firmness and rancidity [20]. IV is an established method utilized by the meat industry to determine the degree of unsaturation. IV can be measured by two different methods. IV values are predicted by the iodometric method such as the Wijs method and Hanus method, or calculated from the fatty acid profile. The iodometric method is based on the reaction of FAs with iodine which results to the addition of the iodine at the double bond in a carbon-carbon chain. The unreacted iodine is converted to molecular iodine by potassium iodide which is titrated against a standardized sodium thiosulphate to determine the amount in grams of iodine consumed per 100 g fat. The higher the IV, the greater the number of double bonds and hence the corresponding higher level of unsaturation in the fat. However, the accuracy of these methods depends on certain experimental parameters such as temperature, accurate timing, and shield of the reacting chemical mixture from light [21]. For measurement based on calculation, IV is estimated by first using gas chromatography (GC) to obtain the FAs, which are further utilized in equations to get the IV [22]. Although the prediction of IV by the calculation method shows more accuracy than the titration methods, in any case, they involve the use of highly toxic and environmentally unfriendly chemicals which require the need for an objective, safe, rapid, and low-cost alternative techniques.

### 2.3. Solid Fat Content

The consistency of fat could be defined regarding firmness and SFC. Firmness has been measured by several instrumental techniques for instance by a penetrometer [23], a texture analyzer [24] and Instron materials testing machine [25]. Davenel, et al. [26] measured the firmness of pork fat and indicated that the puncture test was prone to error if the backfat thickness was less than 12 mm. The authors proposed NMR test as an alternative. SFC is the percentage of solids in fat at specified temperatures. It is an important feature that influences appearance, flavor release, melt rate, shelf life and stability of fat based food products. SFC is particularly of primary importance when considering fat quality because soft fats could be difficult to dimension and process thus causing the decrease of high-quality cuts that could lead to monetary loss of value. Furthermore, in the package, soft fats will have an oily appearance that is unpleasant to the consumer. The softness or oiliness of fat depends on the FA composition which also affects the SFC of pork fat at any given temperature. SFC is reported to increase in the order *cis*-MUFA < PUFA < SFA [27]. Davenel, Riaublanc, Marchal and Gandemer [26] indicated that the melting point of lipid and the firmness and hardness of carcass fat is closely related to the concentration of stearic acid (18:0) and palmitic acid (C16:0), and it has a linear relationship with IV at 20 °C. In the meat product industry, it is desirable to manufacture products with the ideal SFC that will allow for the product to remain solid at room temperature, but still, give consumers the desired mouth-feel experience. Knowing various characteristics of a product from SFC provides an important quality control parameter for the product in a way that achieves the highest quality product.

The traditional methods have been used for routine analysis for the measurement of SFC. Dilatometry is the oldest, and the results are not accurate [28]. Newer techniques such as NMR and differential scanning calorimetry are now useful methods for evaluating SFC. NMR measurement data are calculated based on the relative number of protons present in the triglycerides in the solid and liquid phases, while DSC values are obtained by the melting enthalpies of these triglycerides [29]. For several years, NMR has been the method of choice for the determination of SFC. Although, NMR techniques are rapid, non-destructive, and rarely require re-calibration over a long-term measurement, sample conditioning such as tempering is needed before the measurement is made at each required temperature. On the other hand, DSC provides a means of tempering the fat at various temperatures before measurement. It has the advantage of providing a thermogram including all the temperature range from a single measurement from which the SFC can be obtained from the partial integration of the thermogram [30]. Besides easy operation, DSC provides information about the thermal transitions that the fat may undergo during processing since adding fat to a product without the desired melting profile could cause encapsulation of other ingredients [31]. In fact, DSC measurement of melting characteristics is an internationally accepted conventional method by the American Oil Chemists Society (AOCS, Cj 1–94).

Several reports have emerged over the years regarding SFC results analyzed by NMR and DSC methods, with higher values been yielded by the DSC [28,32,33]. Some authors have attributed these differences to tempering methods and weight of the sample [34]. Others argued that these differences are as a result of calculating NMR data based on the relative numbers of protons present in the triglycerides in the solid and liquid phases, while DSC values are determined based on the melting enthalpies of these triglycerides [28]. Considering that, measures have been taken to correct for the differences [29]. In any case, NMR and DSC still require sample preparation and cannot be applied for rapid assessment of pork SFC. Therefore, there are still efforts being made to find more suitable methods for determination of SFC. A reliable SFC measurement in pork processing plants requires novel, rapid, non-destructive techniques to overcome the limitations of the current instrumental techniques.

### 2.4. Color

One of the most notable features of pork to consumers is its appearance, and the fat color is an important attribute since it is a visible feature by which consumers judge the quality of meat. Since the discoloration of meat often indicates unwholesomeness, consumers evaluate meat product quality by its appearance which in turn influences their buying decision. The fat color is a complex topic involving animal genetic, nutrition, age, sex, fat depot, ante- and post-mortem conditions [35]. Moreover, several other factors related to processing such as packaging, distribution, storage, display, and final preparation for consumption affects meat color. Yellow fat in pork carcasses is less acceptable than white fat, and meat containing yellow fat is sold at lower prices. Yellowness of fat is due to the presence of carotenoid pigments within adipocytes or certain diseases. Beta-carotene is the main contributing carotenoid pigment in pork fat although trace amounts of alpha-carotene and xanthophylls have also been found.

Many investigations on fat color have used human assessors or laboratory methods to measure carotenoid concentration in a sample of excised fat some of which depend on the extraction of pigments from food products followed by spectrophotometric determination of pigment concentration. However other simpler methods consist of combinations of percentage reflectance values and tristimulus values. Color measurements through interaction of light are usually done using the Commission International De I’ Eclairage (CIE) color system, that provides the reference standard CIELAB or CIEXYZ color spaces (Commission International De I’ Eclairage, 1978; Agullo’ et al., 1990; Yam and Papadakis, 2004; Feiner, 2006). Presently, several other options such as colorimeters and spectrophotometers exist. Each instrument offers a variety of choices that allow selecting from various color systems.

Many color spaces have been employed in the assessment of fat color. Hardware-oriented color space such as Red Green Blue (RGB), and Cyan Magenta Yellow (Black) (CMY(K)) have been employed [36]. Also, human-oriented space CIEL∗u∗v∗, and CIEL∗a∗b∗, which considers human sensory perception has been used in pork [37] by which the meat color was specified. In the CIE L*a*b* color space, L* represents the surface lightness/darkness, a*, the redness/greenness and b*, the yellowness/blueness. Thus, the b*- value is considered the ideal objective measurement of the yellowness of a fat surface [37]. In subcutaneous fat CIEL*a*b* variables significantly affect color with L* and chroma being the most affected. L* value correlates well with the FAs composition [38]. The common and simplest way of measuring fat color is usually by comparing the color of the fat and scoring against the fat color reference standard. Though color assessors are well trained for assessing fat color, the subjective nature of the assessment means that there can be variability in the grading scores between the assessors. Consequently, reliable methods, preferably rapid and non-invasive techniques, could be utilized to evaluate critical parameter such as fat color to ensure high-quality pork meat.

### 2.5. Oxidative Degradation

Lipid oxidation in food constitutes a complex chain of reactions [39]. It occurs due to exposure of the labile lipids components to oxygen. The FA composition of adipose tissue influence the tendency to oxidative degradation [40]. Lipid oxidation produces primary products (hydroperoxide) that further decomposes to yield various low-molecular-weight secondary compounds including aldehydes, ketones, and alcohols [41]. These compounds are responsible for the development of rancidity, off-flavor, off-odors and color changes that can be perceivable by consumers [39,42]. Furthermore, they have health effects [43], therefore, lipid oxidation is a critical factor that has a considerable influence on the loss of meat freshness that may lead to both commercial and health consequences. Therefore, its control is a fundamental issue. Several traditional methods for monitoring lipid oxidation in foods have been reviewed by several authors [39,44,45,46].

Lipid oxidation commonly leads to primary and secondary changes. Primary changes involve measurement of the hydroperoxide such as peroxide value (PV), while secondary changes are based on the measurement of the decomposed products of hydroperoxide such as MDA, pentanal, propanal, and hexanal [39]. Although various compounds are produced during the decomposition of hydroperoxide, malondialdehyde (MDA) and hexanal are the most important and abundant aldehydes used as oxidation indices [47]. Thiobarbituric acid reactive substances (TBARS) tests are commonly used to determine the extent of the formation MDA in foods [48]. Gray and Monahan [44] stated that TBA tests are simple to use, and offers considerable sensitivity and versatility for detecting the occurrence of lipid oxidation and other radical reactions but the interpretation of the result should be made with caution. The authors recommended for combined parameters such as hexanal levels when assessing lipid oxidation. In a similar opinion, Laguerre, et al. [49] noted that measuring the extent of oxidation with just one or two markers is a rather coarse approach, so methods involving assessment of a large set of compounds should be encouraged. Fortunately, despite the complex nature of lipid oxidation, with the introduction of advanced chemometric methods, it could be possible to study the multi-parameters of oxidation such as PV, AV, TBARS, and hexanal using modern analytical techniques for developing models for their prediction since the current chemical methods, although accurate, are time-consuming.

## 3. Non-Destructive Spectroscopic Techniques

This section describes the principles and applications of non-destructive techniques including infrared spectroscopy, nuclear magnetic resonance, Raman spectroscopy and hyperspectral imaging. The multivariate data analysis which has been employed for the assessment of fat quality are also discussed. The main general advantages and drawback of each non-destructive technique are presented in Table 1.

### 3.1. Infrared Spectroscopy

Infrared spectroscopy or vibrational spectroscopy includes several different techniques, the most important and commonly applied in food are mid-infrared (MIR), and near-infrared (NIR) [10]. IR spectroscopy measures the broad overtone and combination bands of some of the fundamental vibrations and is an excellent technique for rapid, accurate quantitation and classification. The infrared spectrum originates from radiation energy that is transferred to atoms held together in a chemical bond by molecules in a material. At ambient temperature, the molecules exist at their fundamental vibrational levels, and the molecules are being displaced about one another, which depends on the masses of the atoms, their geometric arrangement, and the strength of their chemical bonds [50,51]. The vibrational amplitudes of these chemical bonds are a few nanometers which would increase if energy is increased. In spectroscopic measurement techniques, this energy is provided by the illumination system which provides the electromagnetic radiation that interacts with the food. Food tissues, as with all biological materials, are composed of different molecular bonds such as O-H, C-H, N-H, and C=O. These bonds contain feature information about the physical and chemical composition. By IR spectrometers in combination with chemometrics, the residing information is obtained indirectly and non-invasively [52].

IR Spectrometers are usually divided into wavelength range, namely, a short wave near-infrared spectral region (SW-NIR) of 780–1100 nm, and long wave near infrared spectral region (LW-NIR) of 1100–2526 nm [53]. In the NIR spectral region, the overtones and combinations of fundamental vibrational responses occur which contains feature information from the chemical bonds (Tan et al., 2012). NIR instruments work on the principle of absorption, reflection, transmission, and scattering of light in or through a food material following the Beer-Lambert law. Absorption or reflectance of light in the known range of wavelengths is used to measure and correlate with an analyte of interest in the food. Therefore NIRS has established itself as a useful analytical technique in the food industry. With data mining and processing based on the chemometric methods, IR spectroscopy has an extensive range of applications and hence provides solutions to several important and challenging analytical problems in a rapid and non-invasive manner which would thus be a valuable alternative technology for measuring the constituents of food. NIRS has been used in pork fat quality assessments for the development of quantitative models for FAs and the FA classes including SFA, MUFA, and PUFA. Overall, most of the studies utilized subcutaneous fat with samples analyzed either in homogenized or intact forms.

The specific absorption of C-H bonds of FA at 1100–1400, and 2200–2400 nm [54] explains the satisfactory performance of NIR predictability of FAs. Most studies have focused on building prediction models that are based on the FAs, and IV. In many cases, the FAs were predicted using modified partial least square regression (MPLSR) from the NIR data pre-processed with different methods (Table 2). In MPLSR, the residuals at each wavelength are obtained after each factor is calculated by dividing the standard deviations of the residuals at a particular wavelength before calculating the next factor [55]. Regarding individual FAs, NIRS has indicated prediction accuracies in both homogenized and intact samples of adipose or meat tissues with an average coefficient of determination ranging from 0.66–0.87 and prediction errors from 0.01–9.08% (Table 2). As Table 2 shows, palmitic acid (C16:0), stearic acid (C18:0), oleic acid (C18:1) and linoleic acid (C18:2) yielded better predictions. The poor prediction obtained from the rest of the fatty acids was due to the low concentrations of the FAs in the sample. The coefficients of determinations were improved when prediction models were developed for the FA groups (Table 3) recording a mean R^2^ of 0.90, 0.91, and 0.87 and mean prediction errors of 1.10%, 1.14%, and 1.38% respectively. IV value in pork, an essential fat quality trait, recorded a mean value of R^2^ of 0.91 and error of prediction of 1.27%.

Key factors such as sample presentation, the accuracy of the reference method and the use of samples with enough range of variability of fat component distribution for the calibration are to be considered when developing calibration models for predicting fat quality. In the case of sample presentation, homogeneity within the meat sample influences the accuracy of the estimation of fat quality. Several authors have confirmed that pork fat quality can be accurately predicted in homogeneous samples than in intact samples by using NIRS. Sample presentation has a major influence on the reliability of NIR prediction. Heterogeneity of the meat samples influences the accuracy of the estimation of chemical components. Some authors have suggested that collecting spectra on intact pork could have reduced the prediction accuracy due to muscle fibers absorbing light along their length by a series of internal reflection, and the high-energy absorbance (lower reflectance) of intact samples [56]. On the other hand, homogenization disrupts the original structure leading to an unsystematic arrangement of the muscle fiber as well as averaging the scattering effects by the muscle fibers [57], which yields lower prediction errors. González-Martín, et al. [58] compared the performances of intact and liquid samples of subcutaneous fat of pork using NIRS (1100–2000 nm) and validated the result by using the MPLSR. On intact subcutaneous fat, interval PLSR (iPLSR) was used by Sørensen, et al. [59] to calibrate a NIRS for IV prediction in unmelted fat obtaining high values of coefficient of determination. Foca, et al. [60] also considered the importance of variable selection in the perspective of developing robust models necessary for predicting the IV and FAs of intact pork fat. By the combination of signal processing and iPLSR, a two-step variable selection was applied to select the most informative variables that provide the most correlation with the NIR spectral data. However, the selected variables only achieved an IV and FAs correlation of 0.81 and ≤0.85 with RMSEP of 1.90 and ≤1.24 respectively.

The selection of such wavelengths holds a promise for miniaturization of NIR multispectral spectrometer for cheaper fat quality evaluation Furthermore, since oxidation is a critical issue in fat science, and the temperature contributes to oxidative stability, González-Martín, et al. [61], while trying to estimate the effect of extraction methods on the quality of fat, used NIRS (1100–2498 nm) to predict and compare the effect of solvent extraction and microwave melting on the FAs composition in subcutaneous fat from pork. The microwave extracted fat could not yield all the FA studied. Signal acquisition procedure using NIRS on extracted pork fat sample is not directly implementable in rapid and on-line monitoring systems.

While most of the studies investigated quality in the subcutaneous fat, a study [56], predicted the fat quality of intramuscular fat of pork using NIRS (1100–2000 nm) operated in the reflectance mode. When spectra were recorded on live pigs, carcases, and on samples from the subcutaneous fat with skin, and skin-free subcutaneous fat, and on transverse cut of the subcutaneous layer by means of a high-intensity fibre optic probe NIRS, good results of moderate accuracies were recorded for the major FAs namely 16:0, C18:0, oleic acid C18:1, and C18:2 [69]. Another study [71] on pork product using NIRS reported FA profile of dry-cured minced sausages of spectra collected over the 1100–2498 nm using MPLSR on mathematically pre-treated data using SNV, DT, FD, SD, and their combinations. However, unsatisfactory results were found which could be attributed to the contribution of the ingredients in the sausage in addition to the meat including the mixture of fat and lean from different anatomical parts and even from different animal species.

One of the main challenges when using IR spectroscopic techniques like NIR for prediction of fatty acid composition of complex food matrices is that all calibration models for estimating FAs are simultaneously developed from the same spectrum. Often in these types of data, there is a strong internal correlation pattern between the different FAs, meaning that when the content of one fatty acid is increased from one sample to another, the content of other FAs might be increased or decreased correspondingly. Thus, for many calibrations models built, internal correlations are found to contribute to good prediction models significantly. These internal correlations might be strong or weak, and the only way to ensure developing a robust calibration model is to use external sample validations. As a rule, every model must be validated to test its ability to predict new samples; a calibration model without validation is nonsense [72]. Pérez-Juan, et al. [62] in their study, validated the calibration models with independent samples to check the robustness of the models developed for FAs. The authors used a FT-NIR in the spectral range of 909–2500 nm to estimate FAs of pork from spectral collected from transverse and longitudinal positions of the intact subcutaneous fat of pork ham cut. Data was pre-treated using the EMSC and second derivative after which it was modeled using the PLSR algorithms. Fourier transform infrared spectroscopy (FT-IR) has also established itself as a useful analytical tool for the assessment of fat quality in pork. FT-MIR (2500–20,000 nm) has been used to determined marine FA (C22:5n3 + C22:6n3), IV, MUFA, PUFA, and SFA having yield a coefficient of correlation and RMSECV of 0.970, 0.996, 0.997, 0.993, 0.993 and 0.08, 0.66, 0.40, 0.43, 0.35 respectively, when PLSR was used to develop the calibration model.

One study, involving the measurement of SFC in pork fat demonstrates the rapid assessment capability of NIR spectroscopy (NIRS) technique operated in the 1041–2380 nm spectral range [68]. In this study, the SFC was measured objectively using PLSR on spectra data pre-processed by the first derivative. A promising result for SFC at 0, 10, and 20 °C was estimated with sufficient accuracy yielding a coefficient of determinations and standard errors of prediction of 0.72%, 0.94%, 0.96% and 2.8%, 2.9%, 3.2%, respectively. Although the results were good, the study was conducted on molten pork fat, which requires additional labor for sample preparation. At present, the trend in food analysis is to reduce the drudgeries of tedious sample preparation procedures, which require that intact samples for SFC assessment would be preferred. Furthermore, since this is the only study that has reported the use of NIRS for SFC determination, the paucity of information regarding SFC seems to exist in meat fats not to talk about pork alone. Therefore, there is need to conduct more investigations on this important quality parameter in a non-destructive manner particularly at this time when there are advanced chemometric methods. This same study [68] evaluated the accuracy of prediction models for evaluation of pork fat firmness regarding the penetration force and reported an R^2^ of 0.74 and standard error of prediction of 18.2% in the validation set. However, this error would be considered too high to allow for a reliable prediction of fat firmness.

As the pork industry strive to meet consumer need for high-quality products, quality control techniques are essential for achieving the success. A good alternative could be the infrared spectroscopy which is a rapid and non-invasive technique. Although published results vary considerably, they suggest that NIR is a suitable analytical method to predict the fat quality in pork and pork products. These quality parameters are usually required to make an overall decision on high-quality products. Thus, NIRS could be a method of choice.

### 3.2. Raman Spectroscopy

RS is a branch of vibrational spectroscopy technique that is based on the shifts in the wavelength or frequency of an exciting incident beam of radiation that result from inelastic scattering on the interaction between the photons and the sample molecules. As with many non-destructive technologies, RS techniques has found its way for many applications such as classification, quantification, and safety control of various fruits and vegetables, crops, beverages, meat and dairy products [73]. Furthermore, RS has been used for compositional identification for the detection of adulteration, as well as for basic research in the elucidation of structural or conformational changes that occur during processing of foods [74]. 

Raman spectroscopy has been developed for the detection of fat quality especially in evaluating the fatty acid classes using PLSR in combination with various data pre-treatments (Table 4). Olsen, Rukke, Flåtten and Isaksson [75], pioneered a study to assess the capability of using RS to determine the quality of fat in pork subcutaneous layer. The focus of this study [75] was to evaluate the degree of unsaturation. Raman spectra were measured on melted and intact samples. Routine laboratory analysis using the gas chromatography was used to obtain the reference values which was employed in calibrating the RS. The spectra were pre-treated with the first derivative, and PLSR algorithm was used to develop prediction models from the selected wavelengths (775–1800 cm^–1^, and 2635–3090 cm^–1^) which contain significant peaks related to lipids for evaluating the IV, SFA, MUFA, and PUFA. The result shows that RS showed a good accuracy, evident by the high coefficients of determination. However, the melted fat gave lower prediction errors compared to intact adipose tissue. In another study on backfat, Lyndgaard, Sørensen, van den Berg and Engelsen [76] found a lower prediction accuracy when RS in the spectral range of 200–1800 cm^–1^ was applied to standard normal variate (SNV). The lower accuracy in this study shows the importance of wavelength selection for estimation of fat quality parameters. Essential FAs such as omega-3 and omega-6 are of critical value in body development, and muscle foods such as meat and fish contribute significant amounts in the diets. Attention has been drawn to the fast and rapid methods to assess their qualities properly. Olsen, Rukke, Egelandsdal and Isaksson [77], employed RS to develop prediction models for quantifying omega-3 and omega-6 in adipose tissue by analyzing Raman spectra of pork subcutaneous fat. The study achieved an accuracy of R^2^ of 0.97and 0.91 and prediction error of 0.99 and 0.23 for omega-3 and omega-6 respectively based on PLSR and first derivative pre-treated spectra data and selected wavelengths. Surprisingly, the study showed poor results for the prediction of the ratio of omega-6 to omega-3 in unextracted fat with only a coefficient of determination of 0.31.

In another study on the determination of fatty acids groups using the RS, [78] used the multivariate modeling technique PLSR on the fingerprint region of the spectra for subcutaneous pork fat samples, and found reliable results for prediction of SFA, MUFA, PUFA, and IV attaining a reliable R^2^ with a reasonable root mean square error of prediction (Table 4). Furthermore, individual fatty acids including C14:0, C16:0, C16:1 *CIS*∆9, C17:0, C18:0, C18:1 *cis*∆9, C18:1 *cis*∆11, C18:2 *cis*∆9, 12, C18:1, *cis*∆9,12,15, C120:0, C20:1 *trans* ∆11, C20:2 *cis*∆11,14, C20:3 *cis*∆8,11,14, C20:4 *cis*∆11, C20:1 *cis*∆5,11,14 were predicted with R^2^ of 0.67, 0.89, 0.56, 0.07, 0.72, 0.82, 0.43, 0.90, 0.87, 0.18, 0.46, 0.78, 0.35, 0.60, 0.87 and prediction errors of 0.06, 0.20, 0.20, 0.09, 0.87, 1.3, 0.18, 1.84, 0.22, 0.05, 0.09, 0.10, 0.05, 0.05, and 0.22, respectively. This study revealed the capability of RS for pork fat quality discrimination. In addition, Berhe, Eskildsen, Lametsch, Hviid, van den Berg and Engelsen [78] revealed that the poor prediction of the ratio of omega-6 to omega-3 obtained by Olsen, Rukke, Egelandsdal and Isaksson [77] could likely be due to the modelling of FAs based on the same Raman spectra information arising as a consequence of strong correlation of a less abundant FA to a more abundant FA or their groups (IV, SFA, MUFA, PUFA). In fact, this means that the high coefficient of determination obtained for both less abundant and highly more abundant FAs could be altered or a total FA group. The study reviewed that good prediction model for fatty acid groups could be obtained with RS when PLSR algorithm is applied. It is important to investigate the correlation structure of individual FAs and the degree of unsaturation using other non-destructive spectroscopic methods exhibiting high collinearity in their spectra.

### 3.3. Nuclear Magnetic Resonance

NMR spectroscopy is an important technique that is based on the magnetic properties of the atomic nuclei. NMR is probably the most important technique for finding the structure of organic compounds. The principle of NMR is based on the fact that certain nuclei with odd atomic mass or atomic numbers such as ^1^H, ^13^C, ^19^F, and ^31^P have a property called spin [79]. That means they have a magnetic field like that of a bar magnet [80]. When placed in an external magnetic field this nuclear magnet combines up in one or two ways with or against the applied field. These two different states have slightly different energy levels. Electric, magnetic radiation which has an energy corresponding to the gap between these two states can cause this nuclear magnet to flip from the lower energy to a higher energy. In a ^1^H-NMR spectrum (also known as time domain TD-NMR), as one drops back from the higher energy to a lower energy state, the nuclei of a hydrogen atom in various positions in the molecule gives out energy at slightly different frequencies, and this provides information about the molecule. This energy is picked up by a receiver in a TD-NMR instrument. Two types of NMR are used in meat science: the time domain NMR and spectroscopic NMR. The spectroscopic NMR measures signal versus frequency while the NMR gives relaxation times namely longitudinal relaxation time, T_1,_ and transversal relaxation time, T_2_, which is widely used. The spectroscopic NMR provides peaks at given frequencies that correspond to molecules of the sample under assessment. The two NMR methods (time domain and spectroscopy) can be performed not only on the protons but also for other nuclei containing odd numbers of protons or neutrons [79].

An important industrial application of ^1^H-NMR spectroscopy has been proposed regarding the assessment of vegetable and animal fats quality. Several studies have employed this technique to provide information about the fatty acid profile, lipid classes, unsaturation level, oxidative stability, and SFC in edible oils and fat [28,32,34,79,80,81]. SFC is an important parameter for the pork industry, and NMR is the most commonly used method for its assessment. The time domain NMR (TD-NMR) has been widely applied and widely accepted as ISO and AOAC method. SFC evaluation by TD-NMR experiments used either the direct or indirect methods, which differ based on the relaxation rates of the protons in solid and liquid fat. The protons in the solid fat tend to relax faster to their equilibrium state after excitation than those in the liquid fat. Other methods are based on the different relaxation rates of protons in the solid and liquid fat. In pork, one study [81] was conducted to exploit the potential of evaluating the acyl chain composition of the lipids of dry-fermented salami quantitatively using NMR. The common unsaturated FA chains present in the lipid fractions of pork was determined using the NMR technique. Furthermore, NMR could determine the total amount of saturated FA as well as the ratio of saturated to unsaturated, and polyunsaturated to saturated which are the two most important criteria determining the nutritional quality of pork meat. NMR showed itself as a powerful alternative to the GC method for determination of the FA chain composition in pork meat. Given the non-invasiveness of NMR technique, and the fact that both biochemical (spectroscopy) and spatial information (imaging) (nuclear magnetic imaging, MRI) can be currently obtained without destroying the sample is obviously a great asset for more studies of the technique for pork fat quality assessment.

## 4. Hyperspectral Imaging Analysis for Fat Quality Assessment

One recent advancement and significant development in non-destructive spectroscopy is the application of hyperspectral imaging techniques. Th hyperspectral imaging camera collects information of a sample across the electromagnetic spectrum. There are three basic methods by which HSI can be used to acquire images of food. These include the point-to-point, line-by-line, and area scan methods. These methods are base on the relative movement of the sample and the camera. In the point-to-point scan method, a single point is scanned by moving either the sample or the detector. By extension of the point-to-point method, the line-scan method (i.e., the push-broom method) simultaneously acquires a spatial information as well as full spectral information for each spatial point in the image. A 2-D image (y, λ), with one spatial dimension (y) and one spectral dimension (λ), is taken at a time. A complete hypercube obtained as the slit is scan in the direction of motion (x). In these methods, usually, the products are placed on the conveyor belt and are moved passed the sensor. HSI systems that are designed and equipped with imaging spectrographs work in the line-by-line scan mode. The line-by-line scanning is more suitable for use on the conveyor belt since a continuous scanning in one direction is its principal characteristic. However, for whatever mode used to obtain images, the overall aim is to get information (spectral data) for every pixel in the image. These data represented by matching spectrum measures the values of the intensities of the reflectance at all wavelengths.

**Figure 2:**
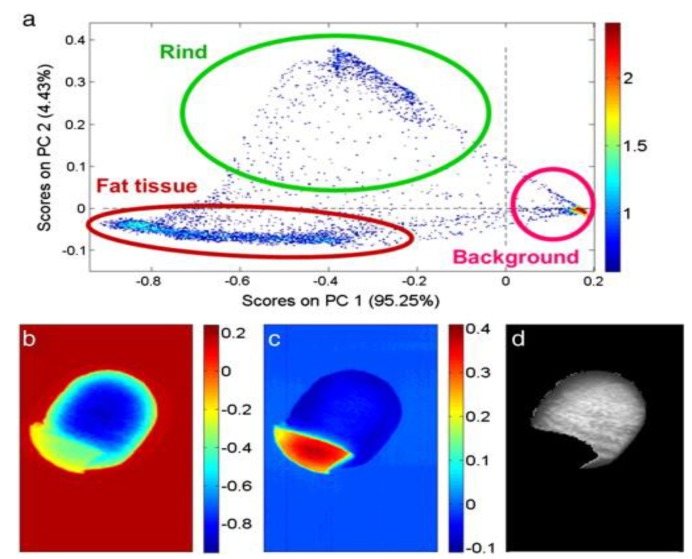
Classification of pork sample from the into rind, adipose tissue, and background (**a**); The background (**b**) and fat tissue (**c**) pixels. (**d**) is the gray image of the pork sample [82].

In the pork industry, the classification of fat into distinct attributes is necessary for selecting the end use. Research work related to pork classification using hyperspectral imaging has been reported in the NIR region. Foca et al. [82] verified the suitability of NIR hyperspectral imaging to discriminate between the outer and inner layers of pork subcutaneous fat. Partial least square-discriminant analysis (PLS-DA) was used to develop the classification models using full wavelengths and selected wavelengths based on these layer classes. The performance of the classification model was expressed regarding efficiency, which was defined as the geometric mean of the percentage of objects of inner or outer layer correctly accepted by the class model, and the percentage of objects of another class correctly rejected by each class model. The results showed that HSI correctly classified pork subcutaneous fat with an accuracy of 99.8% and 99.4% using full wavelength range and selected wavelengths, respectively. In the same study, PCA was used to classify rind, fat tissue, and the background from the pork samples (Figure 2).

The prediction and visualization of the chemical composition in meat using near-infrared HSI and multivariate analysis are the interesting studying field. HSI has been exploited by many researchers [83,84,85,86,87,88,89] for predicting fat content in pork. The next step in this field is not only to quantify the fat content but to develop a rapid method to characterize the quality of the fat. It is worth mentioning that major constituents are easier to predict than minor constituents such as FAs. In pork cuts and their processed products, the fat concentration and composition may be heterogeneously distributed. To understand the distribution, spatial information is valuable. Thus, an imaging-based method which non-invasively provides the fat quality on a continuous scale in each image pixel could be useful.

**Figure 3:**
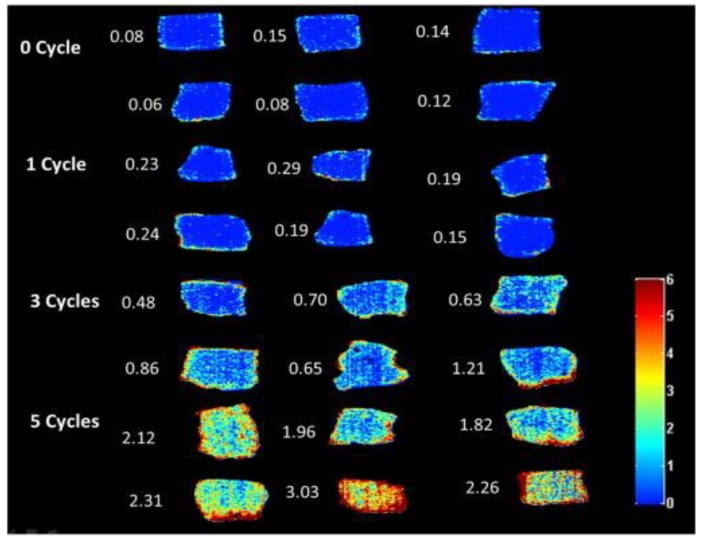
Chemical maps of TBARS distribution in pork samples by different frozen–thawed cycles [91].

Oxidative degradation processes involve many complex red-ox reactions, and a variety of lipid oxidation products are formed. It is the main factor limiting shelf life in many food products [90]. Therefore, the study of the oxidation could indicate the quality of the fat. Traditionally, lipid oxidation is monitored by using chemical analysis to measure some critical oxidative parameters such as malondialdehyde (MDA) (one of the most significant products of oxidative degradation) which is quantified by thiobarbituric acid reactive substance (TBARS) tests. Recently, NIR HSI (874–1734 nm) was applied for developing calibration models for quantification and mapping of TBARS value distribution in frozen-thawed meat for pork samples assigned to four different frozen-thawed cycles (0, 1, 3 and five frozen-thawed cycles) [91]. After hyperspectral image acquisition, TBARS value of each sample was measured, and a calibration model was developed based on PLSR from featured wavelengths selected by successive projection algorithm (SPA). The result shows that the predicted TBARS yielded a coefficient of prediction in the prediction set of 0.67 and a RMSE of 0.33 MDA/kg with input of 13 most important wavelengths (1150, 1355, 1386, 1130, 1072, 1009, 1463, 1328, 1409, 1029, 1598, 1106 and 1214 nm,) selected by the SPA. The developed PLSR model was applied in a pixel-wise manner to produce chemical images showing the amount and distribution of fat degradation in pork under a frozen-thawed cycle (Figure 3). Although the authors successively showed the chemical map to indicate the heterogeneity of TBARS distribution, the developed model still need improvement for a reliable prediction of TBARS. The authors attributed the moderate R^2^ to the rather uncertainty in the chemical methods and measurement units of TBARS measurements, which perhaps affected the accuracy of the chemical maps. Indeed, building chemical maps depends on the precise calibration of the model otherwise, the model would create misleading distribution maps that would be erroneously interpreted [92]. Another study in this area had shown that proper mathematical treatment of the spectra data improved the results when Vis/NIR HSI was used [93]. This improvement could probably be due to the inclusion of data from the visible region since myoglobin such as oxymyoglobin, deoxymyoglobin, and metmyoglobin gives rise to colors which absorb in the visible range [57,94]. Overall the results indicated that NIR HSI combined with image processing has the potential to estimate the degradation in pork fat.

As for other fat quality parameters including FAs, SFC, and color, there are no studies conducted to investigate the ability of HSI technique for their estimation despite being important quality attributes of pork and pork product. Therefore, relevant studies need to be developed for assessing these parameters. It is worth mentioning that the potential of HSI (1000–2300 nm) for predicting individual FAs and FA groups in beef meat has been successfully investigated by [95]. The prediction of SFA and UFA were satisfactory with R^2^, standard error of prediction (SEP) and the ratio of prediction to deviation (RPD) values of 0.87 and 0.89, 1.69% and 3.41%, and 2.43 and 2.84, respectively. For the individual FAs, the R^2^ and RPD values ranged from 0.68–0.89 and 1.69–2.85, respectively. However, for some individual FAs (C14:0, C16:0, C18:0, C14:1, C16:1, C18:1 and C18:2), the result of the R^2^ was rather low. This low R^2^ result is possible because the same regression model is not able to predict various properties of individual FAs [96]. Furthermore, the study revealed that HSI could successively show FAs distribution in a chemical map. This study has proved that HSI is an effective and advanced method for predicting the fatty acid content of meat. However, this is the only study that has demonstrated the ability of HSI for non-destructive and rapid assessment of the FAs composition of meat. More studies would be required to confirm the result of this study in other meat products such as pork. The challenging task of fat oxidation measurement demands more than one method to evaluate lipid degradation in meat. Therefore, choosing just one parameter to analyze the oxidative status is rather challenging, and it is frequently more convenient to combine different methods. The approach of determining oxidative status in a complex matrix of chemical components such as meat is new and further investigations of several parameters are required.

## 5. Future Studies

Research on analytical techniques has considerably evolved since the early 1900s, developing from the dependence on basic ‘wet chemistry’ laboratory methods at the beginning of the century to their gradual replacement by modern instrumental techniques by the start of the twenty-first century. The increasing incorporation of different types of instruments for the analysis of chemical properties such as hand-held, portable and rugged instruments, spectrophotometry, chromatography and separation methods, and the development of process analytical technologies (PAT), amongst others, had an immediate effect on the development and improvement of different food analysis applications. Also, the advances in these methodologies have determined significant improvements in analytical accuracy, precision, detection limits and sample throughput, expanding our capability of analysis into different food matrices and applications. The development, growth, and incorporation of food systems in the global economy greatly relies on better and improved methods for the analysis of foods, outside the simple characterization using few chemical parameters. The use of different tools or methodologies allows for the development of new products, quality control processes, regulatory enforcement activities and problem-solving tasks that are based on the application of modern statistics concepts, experimental design, and chemometrics where they become an integral part of the analytical process and analysis of foods. Food scientists all over the world are dealing with massive amounts of data derived from different measuring devices, sensory experiments, and processes. Therefore, the meat industry must take a step further to utilize additional tools for data analysis to increase the accuracy of prediction of fat quality by making use of the present chemometric methods. For instance, the use of non-linear methods such as least square support vector machines (L-SVM), and neural networks (NN) especially deep learning methods could be applied.

The result of the previous research works presented in this review confirmed that non-destructive spectroscopic techniques are well suited for predicting the quality of fat in pork such as SFC, FAs, color, and oxidative stability. Consequently, the chemical methods currently used by the meat industry can be replaced by the non-destructive techniques. This review has indicated that NIRS and RS have been used extensively for the assessment of fat quality, especially the FAs. However, despite their vital importance in meat quality processing, these technologies have not been exploited for other fat quality parameters in pork. The focus presently is on the evaluation of fat content rather than its quality even though these technologies have shown the potential for evaluating fat quality. As the search for novel techniques for food quality evaluation continues, there is a need to turn to the non-destructive and remote sensing capability of HSI for food quality evaluations in general, and the pork industry in specific. Moreover, there is a need to utilize the potentials of HSI for evaluating minor chemical components. It is expected that HSI will be exploited soon for monitoring more complex parameters of pork fat quality such as FAs, SFC, and multi-parameters of fat oxidative degradation, for on-line applications. HSI provides an interesting platform for meat quality monitoring and control with the chemical and physical information residing in the huge amount of spatial and spectral data.

## 6. Conclusions

This review has mainly focused on four spectroscopic techniques including IR spectroscopy, RS, NMR spectroscopy, and HSI in the aspect of assessing SFC, oxidative degradation, color and consistency in pork and pork products fats. NIRS and RS have been extensively used in fat quality investigations in pork especially for FA assessment, and they have shown great ability to provide accurate information. However, not so much efforts have been made in the evaluation of other quality parameters. On the other hand, the use of NMR spectroscopy, confirmed by several research groups, has seen very satisfactory for measuring fat quality. As for HSI, it is an emerging technique that integrates the merits of spectroscopy and computer vision and has shown to be a valuable tool for pork fat content, TBARS evaluation. However, studies in utilizing HSI for SFC, IV, and FA in pork have not been conducted. As pork products manufacturers develop new food products, a focused on the multidisciplinary approach to assessing fat quality could be important in its success. Recently, researchers and analysts are looking for optimal wavelengths at which robust models can be built to measure the characteristics associated with a meat quality attribute accurately. By understanding these critical wavelengths, more simple and low-cost instruments using only these wavelengths can be introduced to the pork industry.

## Figures and Tables

**Table 1:** Advantage and drawbacks of non-destructive spectroscopic techniques for fat quality assessment.

Technique	Advantages	Drawbacks
IR	Low instrument cost No special skills are required for operation Provides multicomponent information	Requires complex data analysis Prediction success depends on reliability of reference method No spatial information
Raman	Simple to operate Provides spectral information Provides multicomponent information Sensible to minor component detection	Not suitable for materials that emit strong fluorescence Require complex data analysis Strong interfering with biological fluorescence background signals Laser heat could affect the effectiveness of assessments
NMR	Presence of spatial information Provides multicomponent information Fat quality information can be obtained in vivo	Highly expensive equipment Strict testing environment Requires specific skills to interpret the spectra Insensitive to minor fat component detection
HSI	Provides spatial and spectral information that shows component distribution Provides multicomponent information Sensible to minor component detection Relatively easy to operate Insensitive to minor fat component detection	Requires complex data analysis Prediction success depends on reliability of the reference method High instrument cost Redundancy of information in the hypercube Requires time and skills to acquire the desired information from the spectral images

**Table 2:** Prediction of individual FAs in pork by Near infrared spectroscopy.

**Spectral Range (nm)**	**Sample Presentation**	**C12:0**		**C14:0**		**C16:0**		**C17:0**		**C18:0**		**C20:0**		**C20:1**		**Test Set Samples**	**Data Prep. Method**	**MVA Method**	**Ref.**
		**R^2^cv**	**SEP**	**R^2^cv**	**SEP**	**R^2^cv**	**SEP**	**R^2^cv**	**R^2^cv**	**SEP**	**R^2^cv**	**SEP**	**R^2^cv**	**SEP**	**R^2^cv**				
1100–2000	Loin (I)	0.62	0.01	0.79	0.14	0.80	1.10	0.83	0.03	0.77	0.93	0.46	0.04	0.64	0.10	No test set	MSC, DT, SNV, 2D	MPLSR	[56]
1100–2000	Sub. Fat (I)	-	-	0.67	0.10	0.94	0.58	-	-	0.87	0.80	-	-	0.54	0.24	No test set	SNV, DT, 1D, 2D	MPLSR	[58]
1100–2500	Sub. Fat (H)	0.84	0.01	0.70	0.11	0.89	0.66	0.62	0.04	0.85	0.54	0.85	0.03	0.66	0.23	No test set	MSC, SNV, DT, 1D, 2D	MPLSR	[61]
900–2500	Sub. Fat (H)	-	-	0.75	0.20	0.37	1.40	-	-	-	-	-	-	-	-	No test set	EMSC, 1D, 2D	PLSR	[62]
400–2500	Sub. Fat (H)	-	-	-	-	0.87	0.38	-	-	0.78	0.36	-	-	-	-	No test set	NOR, 1D, 2D, MSC	MPLSR	[63]
400–2500	Sub. Fat (H)	-	-	-	-	0.97	0.27	-	-	0.99	0.32	-	-	-	-	Test set	SNV, DT	MPLSR	[64]
400–2500	Sub. Fat (H)	-	-	-	-	0.84	0.87	-	-	0.96	0.64	-	-	-	-	Test set	MSC, SNV, DT	MPLSR	[65]
400–2500	Sub. Fat (I)	-	-	-	-	0.92	0.45	-	-	0.95	0.36	-	-	0.67	0.07	Test set	1D, 2D	PLSR	[66]
400–2500	Sub. Fat (H)	-	-	-	-	0.87	0.79	-	-	0.90	0.45	-	-	-	-	No test set	1D, 2D	PLSR	[66]
400–2500	Sub. Fat (I)	-	-	-	-	0.80	0.88	-	-	0.70	0.80	-	-	-	-	Test set	MSC, SNV, DT, 1D, 2D	PLSR	[67]
1600–2400	Sub. fat (I)	-	-	-	-	0.78	1.00	-	-	0.83	0.68	-	-	-	-	Test set	1D, 2D	PLSR	[66]
	Sub. Fat (I)	-	-	-	-	0.42	1.12	-	-	0.59	1.37	-	-	-	-	Test set	SMO, DT	iPLSR	[60]
1042–2380	Sub. Fat (H)	-	-	-	-	0.88	1.70	-	-	0.94	2.20	-	-	-	-	No test set	1D, NOR	PLSR	[68]
450–2300	in vivo	-	-	-	-	0.74	1.24	-	-	0.72	0.67	-	-	-	-	No test set	SNV, DT, 1D, 2D	PLSR	[69]
450–2000	Carcass	-	-	-	-	0.87	0.82	-	-	0.46	0.94	-	-	-	-	No test set	SNV, DT, 1D, 2D	PLSR	[69]
450–2300	Fat with skin	-	-	-	-	0.86	0.89	-	-	0.80	0.57	-	-	-	-	No test set	SNV, DT, 1D, 2D	PLSR	[69]
450–2300	Fat without skin	-	-	-	-	0.88	0.81	-	-	0.80	0.57	-	-	-	-	No test set	SNV, DT, 1D, 2D	PLSR	[69]
1100–2300	Transverse	-	-	-	-	0.93	0.65	-	-	0.84	0.54	-	-	-	-	No test set	SNV, DT, 1D, 2D	PLSR	[69]
**Mean**		0.73	0.01	0.73	0.14	0.81	0.87	0.73	0.04	0.81	0.75	0.66	0.04	0.63	0.16				
**STD**		0.11	0.00	0.05	0.04	0.16	0.35	0.11	0.01	0.13	0.44	0.20	0.01	0.05	0.08				
**Spectral Range (nm)**	**Sample Presentation**	**C16:1**		**C17:1**		**C18:1**		**C18:2**		**C18:3**		**C18:2n-6**		**C18:3n-3**		**Test Set Samples**	**Data Prep. Method**	**MVA Method**	**Ref.**
		**R^2^cv**	**SEP**	**R^2^cv**	**SEP**	**R^2^cv**	**SEP**	**R^2^cv**	**SEP**	**R^2^cv**	**SEP**	**R^2^cv**	**SEP**	**R^2^cv**	**SEP**				
1100–2000	Loin (I)	0.79	0.47	0.76	0.03	0.70	1.09	0.86	1.15	0.88	0.10	-	-	-	-	No test set	MSC, DT, SNV	MPLSR	[56]
1100–2000	Sub. Fat (I)	-	-	-	-	0.89	1.19	0.95	0.52	0.61	0.13	-	-	-	-	No test set	MSC, DT, SNV	MPLSR	[58]
1100–2500	Sub. Fat (H)	0.75	0.24	0.66	0.04	0.91	1.15	0.88	0.49	0.77	0.12	-	-	-	-	No test set	MSC, SNV, DT, 1D, 2D	MPLSR	[61]
900–2500	Sub. Fat (H)	-	-	-	-	-	-	-	-	-	-	0.68	1.10	0.38	0.10	No test set	EMSC, 1D, 2D	PLSR	[62]
400–2500	Sub. Fat (H)	-	-	-	-	0.86	0.59	0.91	0.23	-	-	-	-	-	-	No test set	NOR, 1D, 2D, MSC	MPLSR	[63]
400–2500	Sub. Fat (H)	-	-	-	-	0.99	0.20	0.98	0.16	-	-	-	-	-	-	Test set	SNV, DT	MPLSR	[64]
400–2500	Sub. Fat (H)	0.89	0.10	-	-	-	-	-	-	-	-	0.98	0.29	0.68	0.09	Test set	MSC, SNV, DT	MPLSR	[65]
400–2500	Sub. Fat (I)	-	-	-	-	0.87	1.19	0.94	0.29	-	-	-	-	-	-	Test set	1D, 2D	PLSR	[66]
400–2500	Sub. Fat (H)	-	-	-	-	1.00	0.4	1.00	0.19	-	-	-	-	-	-	Test set	1D, 2D	PLSR	[66]
400–2500	Sub. Fat (I)	-	-	-	-	0.84	1.15	-	-	-	-	0.83	0.99	0.81	0.22	No test set	MSC, SNV, DT, 1D, 2D	PLSR	[67]
1600–2400	Intact carcass	-	-	-	-	0.83	0.68	0.81	1.30	-	-	-	-	-	-	Test set	1D, 2D	PLSR	[66]
	Sub. Fat (I)	-	-	-	-	0.63	1.39	0.74	0.95	-	-	-	-	-	-	Test set	SMO, DT	iPLSR	[60]
1042–2380	Sub. Fat (H)	-	-	-	-	0.92	1.40	-	-	-	-	0.86	8.70	0.76	35.90	No test set	1D, NOR	PLSR	[68]
450–2300	in vivo	-	-	-	-	0.77	1.42	0.60	0.36	-	-	-	-	-	-	No test set	SNV, DT, 1D, 2D	PLSR	[69]
450–2000	Carcass	-	-	-	-	0.80	1.48	0.31	0.55	-	-	-	-	-	-	No test set	SNV, DT, 1D, 2D	PLSR	[69]
450–2300	Fat with skin	-	-	-	-	0.82	1.44	0.39	0.47	-	-	-	-	-	-	No test set	SNV, DT, 1D, 2D	PLSR	[69]
450–2300	Fat without skin	-	-	-	-	0.92	0.99	0.42	0.58	-	-	-	-	-	-	No test set	SNV, DT, 1D, 2D	PLSR	[69]
1100–2300	Transverse section	-	-	-	-	0.90	1.05	0.64	0.35	-	-	-	-	-	-	No test set	SNV, DT, 1D, 2D	PLSR	[69]
**Mean**		0.81	0.27	0.71	0.04	0.85	1.05	0.74	0.54	0.75	0.12	0.84	2.77	0.66	9.08				
**STD**		0.06	0.15	0.05	0.01	0.09	0.38	0.22	0.34	0.11	0.01	0.11	3.44	0.17	15.49				

PLSR: partial least squares regression; MPLSR: modified PLSR; iPLSR: interval PLSR; R^2^_CV_: determination coefficient of cross-validation; SEP: standard error of prediction; SECV: standard error of cross-validation; H: homogenised sample; I: intact sample; MVA: multivariate analysis; C12:0: Lauric acid; C16:0: Palmitic acid; C17:0: heptadecanoic acid; C18:0: Stearic acid; C12:0: Arachidic acid; C16:1: Palmitoleic acid; C17:1: *cis*- 10-heptadecenoic; C18:1: Oleic acid, C18:2: Linoleic; C18:3: Linolenic acid; C182n-6: Linoleic acid; C18:3n-3: α- linolenic acid, SNV: standard normal variate; DT: detrending; 1D: first derivative; 2D: second derivative; EMSC: extended multiplicative scatter correction; NOR: normalization; SMO: smoothing.

**Table 3:** Prediction of FA groups and IV in pork by NIRS. SFA: saturated fatty acids; MUFA: mono-saturated fatty acids; PUFA: polyunsaturated fatty acids; IV: iodine value.

Spectral Range	Sample Presentation	SFA		MUFA		PUFA		IV		Test Set Samples	Data Prep. Method	MVA Method	Ref.
(nm)		R^2^cv	SEP	R^2^cv	SEP	R^2^cv	SEP	R^2^cv	SEP				
1100–2000	Loin (I)	0.81	1.76	0.94	1.29	0.74	1.25	-	-	No test set	MSC, DT, SNV	MPLSR	[56]
1100–2000	Sub. Fat (I)	0.96	1.10	0.98	1.50	0.95	0.60	-	-	No test set	MSC, DT, SNV	MPLSR	[58]
1100–2500	Sub. Fat (H)	0.92	0.82	0.89	1.12	0.90	0.52	-	-	No test set	MSC, SNV, DT, 1D, 2D	MPLSR	[61]
900–2500	Sub. Fat (H)	0.81	1.70	0.94	1.20	0.73	1.60	-	-	Test set	EMSC, 1D, 2D	PLSR	[62]
400–2500	Sub. Fat (H)	0.95	0.49	0.94	0.65	-	-	0.97	1.22	Test set	MSC, SNV, DT	MPLSR	[65]
400–2500	Sub. Fat (I)	0.86	1.37	0.82	1.23	0.86	1.08	0.87	1.80	No test set	MSC, SNV, DT, 1D, 2D	PLSR	[67]
	Sub. Fat (I)	0.79	1.38	0.77	1.20	0.82	0.85	0.82	1.67	Test set	SMO, DT	iPLSR	[60]
1042–2380	Sub. Fat (H)	0.98	0.90	0.88	1.60	0.96	4.70	0.98	0.80	Test set	1D, NOR, MSC	PLSR	[68]
	Sub. Fat (I)	-	-	-	-	-	-	0.83	1.44	Test set	EMSC	iPLSR	[59]
2500–20,000	Sub. Fat (H)	0.99	0.35	0.99	0.43	0.99	0.4	0.99	0.66	No test set	MSC, 1D, NOR	PLSR	[70]
**Mean**		0.90	1.10	0.91	1.14	0.87	1.38	0.91	1.27				
**STD**		0.08	0.45	0.07	0.335	0.09	1.24	0.07	0.39				

**Table 4:** Prediction of FA groups and IV in pork by Raman spectroscopy.

Sample Presentation	SFA		MUFA		PUFA		IV		SFA	MUFA	PUFA	IV
	R^2^cv	SEP	R^2^cv	SEP	R^2^cv	SEP	R^2^cv	SEP				
Sub. Fat (H)	0.98	0.60	0.92	1.00	0.96	1.00	0.96	1.40	Test set	SNV, 1D	PLSR	[75]
Sub. Fat (I)	0.92	1.10	0.83	1.50	0.90	1.50	0.94	1.80	Test set	SNV, 1D	PLSR	[75]
Sub. Fat (I)	0.50	2.24	0.57	2.28	0.72	1.17	0.69	2.00	No test set	SNV	PLSR	[76]
Sub. Fat (I)	0.84	1.50	0.81	1.53	0.90	1.17	0.89	3.26	No test set	EMSC	PLSR	[78]
Inner Sub. Fat (I)	0.83	1.52	0.80	1.56	0.88	2.29	0.87	3.55	No test set	EMSC	PLSR	[78]
Outer Sub. Fat (I)	0.79	1.69	0.74	1.81	0.85	2.60	0.83	4.10	No test set	EMSC	PLSR	[78]
**Mean**	0.81	1.44	0.78	1.61	0.87	1.62	0.86	2.69				
**STD**	0.15	0.51	0.11	0.38	0.07	0.61	0.09	1.00				

## Nomenclature

ANN	Artificial Neural Networks
AOAC	Association of Official Analytical Chemists
AOCS	American Oil Chemists’ Society
AV	Anisidine Value
DSC	Differential Scanning Calorimetry
EMSC	Extended Multiplicative Scatter Correction
FA	Fatty Acid
FT-NIR	Fourier Transform Near Infrared
GA	Genetic Algorithms
GC	Gas Chromatography
HSI	Hyperspectral Imaging
iPLSR	Interval Partial Least Squares Regression
IR	Infrared
ISO	International Standard Organization
LS-SVM	Least Squares Support Vector Machines
MDA	Malondialdehyde
MIR	Mid Infrared
MLR	Multilinear Regression
MPLSR	Modified Partial Least Squares Regression
MSC	Multiplicative Scatter Correction
MUFA	Monounsaturated Fat
NIR	Near Infrared
NIRS	Near Infrared Spectroscopy
NMR	Nuclear Magnetic Resonance
PCA	Principal Component Analysis
PLSDA	Partial Least Squares Discriminant Analysis
PLSR	Partial Least Squares Regression
PUFA	Polyunsaturated Fat
PV	Peroxide Value
R^2^	Coefficient Of Determination
R^2^c	Coefficient Of Determination In The Calibration
R^2^cv	Coefficient Of Determination In The Cross-Validation
R^2^p	Coefficient Of Determination In The Prediction
RGB	Red, Green And Blue
RMSEC	Root Mean Square Of Calibration
RMSECV	Root Mean Square Of Cross-Validation
RMSEP	Root Mean Square Of Prediction
ROI	Region Of Interest
RPD	Ratio Of Prediction To Deviation
RS	Raman Spectroscopy
SEP	Standard Error Of Prediction
SFA	Saturated Fat
SFC	Solid Fat Content
SNV	Standard Normal Variate
TBARS	Thiobarbituric Acid Reactive Substances
TD-NMR	Time Domain Nuclear Magnetic Resonance
UVE	Uninformative Variable Elimination
